# Building evidence to advance health equity: a systematic review on care-related outcomes for older, minoritised populations in long-term care homes

**DOI:** 10.1093/ageing/afae059

**Published:** 2024-04-01

**Authors:** Mary M Scott, Alixe Ménard, Annie H Sun, Maya Murmann, Amy Ramzy, Prabasha Rasaputra, Michelle Fleming, Zsófia Orosz, Chau Huynh, Vivian Welch, Anna Cooper-Reed, Amy T Hsu

**Affiliations:** The Public Health Agency of Canada, Ottawa, ON, Canada; The Ottawa Hospital Research Institute, Ottawa, ON, Canada; The Ottawa Hospital Research Institute, Ottawa, ON, Canada; Department of Health Sciences, University of Ottawa, Ottawa, ON, Canada; Bruyere Research Institute, Ottawa, ON, Canada; The Ottawa Hospital Research Institute, Ottawa, ON, Canada; Bruyere Research Institute, Ottawa, ON, Canada; Bruyere Research Institute, Ottawa, ON, Canada; Bruyere Research Institute, Ottawa, ON, Canada; Bruyere Research Institute, Ottawa, ON, Canada; Ontario Centres for Learning, Research and Innovation in Long-Term Care, Ottawa, ON, Canada; Bruyere Research Institute, Ottawa, ON, Canada; Ontario Centres for Learning, Research and Innovation in Long-Term Care, Ottawa, ON, Canada; Bruyere Research Institute, Ottawa, ON, Canada; Bruyere Research Institute, Ottawa, ON, Canada; The Campbell Collaboration, Philadelphia, PA, USA; Bruyere Research Institute, Ottawa, ON, Canada; The Ottawa Hospital Research Institute, Ottawa, ON, Canada; Bruyere Research Institute, Ottawa, ON, Canada; Ontario Centres for Learning, Research and Innovation in Long-Term Care, Ottawa, ON, Canada; Department of Family Medicine, University of Ottawa, Ottawa, ON, Canada

**Keywords:** minority health, long-term care, health care quality, access, and evaluation, healthcare disparities, nursing homes, systematic review, older people

## Abstract

**Background:**

Advancing health equity requires more contextualised evidence.

**Objectives:**

To synthesise published evidence using an existing framework on the origins of health disparities and determine care-related outcome disparities for residents of long-term care, comparing minoritised populations to the context-specific dominant population.

**Design:**

Systematic review.

**Subjects:**

Residents of 24-hour long-term care homes.

**Methods:**

The protocol was registered *a priori* with PROSPERO (CRD42021269489). Literature published between 1 January 2000 and 26 September 2021, was searched, including studies comparing baseline characteristics and outcomes in minoritised versus dominant populations. Dual screening, two-reviewer verification for extraction, and risk of bias assessments were conducted to ensure rigour. Studies were synthesized using a conceptual framework to contextualise evidence according to multi-level factors contributing to the development of care disparities.

**Results:**

Twenty-one of 34 included studies demonstrated disparities in care outcomes for minoritised groups compared to majority groups. Thirty-one studies observed differences in individual-level characteristics (e.g. age, education, underlying conditions) upon entry to homes, with several outcome disparities (e.g. restraint use, number of medications) present at baseline and remaining or worsening over time. Significant gaps in evidence were identified, particularly an absence of literature on provider information and evidence on the experience of intersecting minority identities that contribute to care-related outcome disparities in long-term care.

**Conclusion:**

This review found differences in minoritised populations’ care-related outcomes. The findings provide guidance for future health equity policy and research—supporting diverse and intersectional capacity building in long-term care.

## Key points

- Intersecting factors contribute to the persistence of health inequities, particularly in older, minoritised populations.
- Upon entry to long-term care homes at baseline, minoritised populations have differences in characteristics and previously acquired adverse care-related outcomes.
- Quality of long-term care is not uniform and homes with poor quality are more likely to house minoritised populations.
- There is a paucity of literature on factors that impact long-term care outcomes, including provider information, minoritised populations, and their intersection.

## Background

Health inequities experienced by older persons, particularly those belonging to minoritised groups have been well described in the existing literature [1, 2]. Health equity is providing equal and just opportunity for all to obtain their full health potential, while disparities are systematic barriers or a lack of opportunity that prevents a disadvantaged social group from reaching their full health potential [3]. The findings and interpretations derived from the current body of literature tend to be confirmatory—that is, whether differences between demographic and social groups exist—rather than expository. Advancing health equity through research requires highlighting the multiple dynamic layers of individual, group, and system-level factors that intersect and contribute to the experience of disparities—moving beyond unidimensional descriptions of how outcomes differ across minoritised groups [4]. Several theoretical and conceptual frameworks can be applied to elucidate these multi-dimensional and intersecting factors [5–7].

Advancing health equity is important, especially within congregate care settings caring for older, vulnerable populations, such as long-term care homes or nursing homes. Factors that make older adults at risk of inequitable care include their frailty and health vulnerabilities (e.g. a significant proportion of residents in long term care live with physical and cognitive impairments, making self-advocacy challenging), policy-driven and regulated care practices, and an unprecedented worldwide need for system expansion to meet a growing number of older adults with increasingly complex medical needs that are primarily met by private, for-profit providers [8–17].

The World Health Organization (WHO) released an international framework (November 2021) on equitable long-term care, defined as a care continuum that consists of organisations, people and actions [18]. The framework highlighted the importance of leveraging existing systems and experiences to build equitable, person-centred policies. The long-term care sector is a multifaceted and rapidly growing health care sector [19] that provides health, social and residential services to those with chronic disabilities and life-limiting illness [20]. Although significant variation exists across international long-term care systems and definitions [21], care homes providing 24-hour care are a crucial component of the care continuum. Disparities in access to care and health outcomes exist among older adult populations, particularly those who are minoritised in their communities [22–24]. While literature has described improving health disparities in access and outcomes [25–33], there is no synthesis of evidence on the care experiences and outcomes of those living within long-term care homes.

The objectives of this systematic review were to determine and evaluate differences in care-related outcomes (e.g. residents’ symptoms, healthcare use, medical data and quality of life) for minoritised populations in residential long-term care homes compared to more dominant populations receiving care in the same setting, and to contextualise the evidence by synthesizing it within intersecting layers that contribute to the persistence of health inequity.

## Method

### Protocol and registration

A protocol was registered *a priori* with PROSPERO (Protocol ID CRD42021269489). Reporting of methodology and results adheres to the PRISMA-Equity Extension for systematic reviews with a focus on health equity [34].

### Study population

Minority status is an ambiguous term describing either an exclusive social position with advantageous privileges or a disadvantaged position with social vulnerabilities, with the latter more commonly used in existing literature and policy [35, 36]. Minority identity or status can also be understood from the lens of an individual’s own self-perception in relation to their geographic and cultural setting [37]. To accommodate the broad yet context-specific nature of defining minoritised populations, in this paper, we used a definition based on the United Nations’ (UN) Declaration on the Rights of Persons Belonging to National or Ethnic, Religious and Linguistic Minorities (1992), whereby minority status is ‘based on national or ethnic, cultural, religious and linguistic identity’ and dependent on the cultural, geographic, and linguistic area within which each group lives [37]. We also searched for studies about experiences of individuals minoritised for their sexual and gender identity, as they are minoritised and at risk of receiving incongruent care [38]. We will use the term [sic] to indicate the terminology from the original publication, recognizing that the use of the original terms sometimes contributes to a lack of clarity and may be nuanced in intersections and terminology used (e.g. ‘others’, ‘racial differences’, ‘Black’ and ‘Hispanic’).

### Outcome measures

This review defined care-related outcomes as a health-related development or event that may occur or worsen without proper care intervention (e.g. pressure ulcers), an outcome related to care management (e.g. medication use, quality of life) or an event that could indicate a change or disruption in care (e.g. hospitalisation).

### Search strategy and selection criteria

We searched MEDLINE, MEDLINE In-Process, MEDLINE Ahead of Print, Embase, CINAHL, EconLit, ProQuest Dissertations & Theses Global, PsycINFO, Web of Science and the Cochrane Central Register of Controlled Trials for studies published between 1 January 2000, and 26 September 2021. Searches and keywords were restricted to publications in English and French. The search strategy was developed by an information scientist, and peer reviewed in accordance with PRESS guidelines [39]. Non-indexed literature was not included given *a priori* knowledge of the large volume of published studies that already exist within indexed databases, the intent to conduct a meta-analysis of included studies, and because the central research question pertains to findings that explore exposure-outcome relationships found most often in indexed databases [40]. Our full search strategy can be found in Appendix I.

### Inclusion criteria

We included observational and experimental peer-reviewed literature (i.e. trials, cohort, ecological and qualitative designs) on residents in 24-hour nursing care homes (i.e. long-term care, nursing home, residential full-time nursing care facility and care home) who were defined in these studies as a minoritised population or who identified as belonging to a minoritised population. Residents admitted to a long-term care home are routinely assessed as requiring a high level of care need. The homogeneity of individuals needing this level of care across jurisdictions allows comparisons within these populations across published studies. Studies must have compared outcomes of a minoritised population to the context-specific overall population (i.e. cohort studies) or compared homes with higher proportions of minoritised populations to those with lower proportions (i.e. ecological studies).

We excluded studies without stratification of the results by minority status, studies without comparison to the context-specific majority population (apart from qualitative studies) and studies that described minoritised populations without reporting their experience of care-related outcomes. We excluded studies focussing only on outcomes experienced at the time of admission, as it did not allow sufficient time for residents in these facilities to receive routine treatment and support provided within the long-term care home and it would be difficult to attribute their outcomes to the care provided in these facilities. We also excluded studies that did not compare baseline sociodemographic differences between the minority and majority population. This was critical to our research question, as it would be indistinguishable if the observed variation in outcomes or inequities were explainable by heterogeneity in their baseline health or demographic characteristics. Finally, we did not include other systematic or scoping reviews; however, we hand-searched the reference list of all reviews for relevant studies that met our inclusion criteria.

Detailed descriptions of the approaches taken for screening and data extraction, assessment of bias and data synthesis are listed in Appendix II.

### Data coding and analysis

This review applied the conceptual framework of Kilbourne *et al*. [7] to code and narratively summarise results according to the multi-level factors contributing to health disparities. Organising and placing existing literature within a broader conceptual framework can help elucidate knowledge gaps, by providing context without only focussing on individual exposures or single diseases, and ensuring equity is a central feature of the final conclusions [41–43]. Extracted data were coded and organised according to the three dimensions in Kilbourne *et al.’s* framework: Individual factors, provider factors and healthcare system factors.

For individual-level factors, we noted differences in sociodemographic, health and care characteristics between minoritised and non-minoritised populations that were statistically significant (*P* < 0.05) or, if no statistical tests were performed on the summary statistics, we noted characteristics that had a ≥ 1% difference between the comparison groups. Information on provider and healthcare system factors were also extracted and each included study was coded according to: (i) whether provider- or system-level variables were examined and (ii) whether multi-level regression modelling was performed to account for clustering effects. Extracted data were narratively summarised by observed differences in care-related outcomes between minoritised and non-minoritised populations that were attributable to individual, provider and healthcare system factors.

## Results

### Literature search

**Figure 1:**
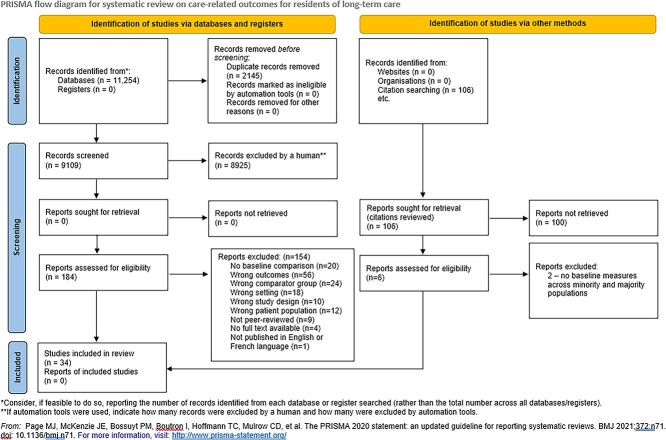
Preferred Reporting Items for Systematic Reviews and Meta-Analyses (PRISMA) diagram for published literature on care-related outcomes of minoritised residents of long-term care homes.

The review is based on 34 articles from a literature search that yielded 9,109 studies and a full-text screening of 184 studies. Manual screening of the reference lists of relevant literature reviews led to four additional studies. Details of the article screening process are outlined in the PRISMA diagram (Figure 1).

### Study characteristics

**Figure 2:**
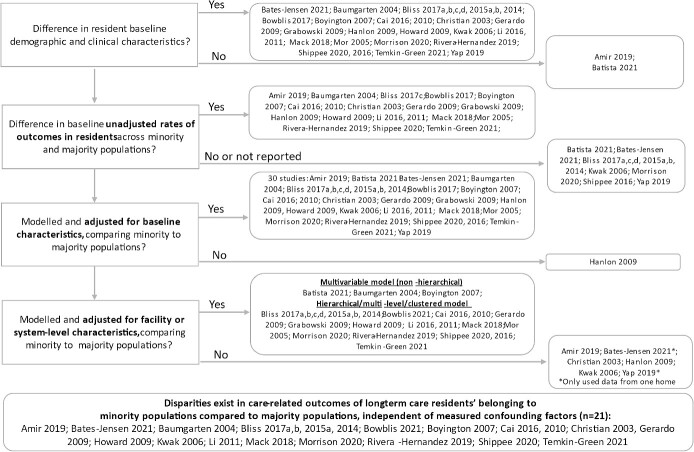
Description of how individual-level and healthcare system-level factors were described and modelled across all included quantitative studies.

**Figure 3:**
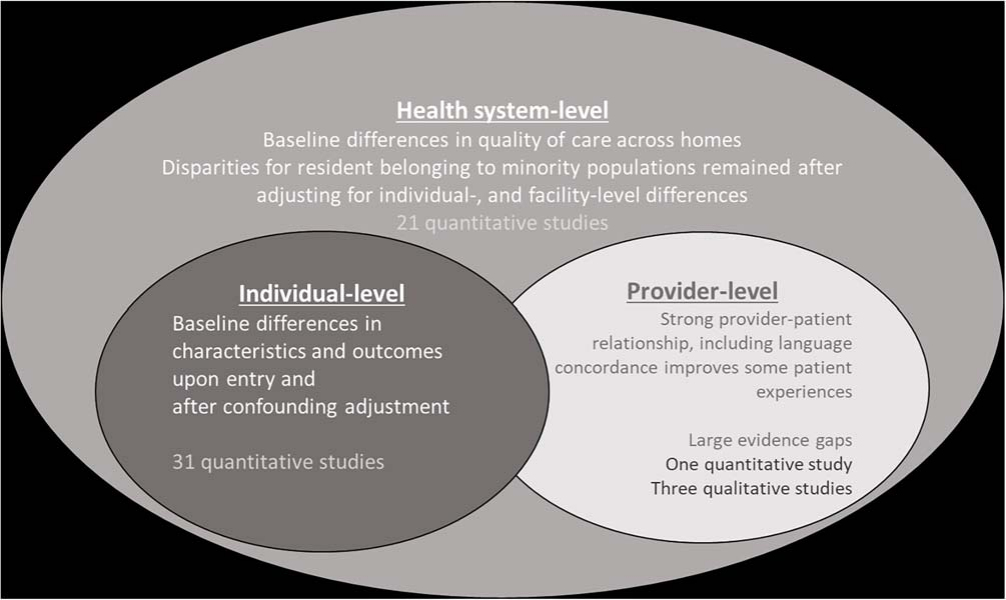
The evidence summarised and mapped on to the domains of the guiding framework. *Framework used to understand the origins of health and healthcare disparities from a health services research perspective: key potential determinants of health disparities within the healthcare system, including individual, provider, and healthcare system factors. Modified from Kilbourne et al. 2006* [7].

The majority (>90%) of the studies were conducted in the United States (U.S.), with two studies from Canada and two from Sweden [44–47]. Over half of the studies restricted resident age to >65 years, three studies restricted to ≥50 years old and nine studies did not report an age restriction, however the average age of each resident cohort was over 65 years. A total of 32 (94%) studies defined minority status using ethnicity or race, while two studies [44, 47] focused on linguistically minoritised populations (Table 1). One study explored French-speaking individuals, who are considered official language minorities in Canada (as English and French are official Canadian languages), and the other was on first languages other than Swedish [44, 47]. All quantitative studies (*n* = 31) used retrospective secondary data sources and described both individual-level and system-level characteristics (Figure 2). Three studies were qualitative studies on the perspectives of residents and caregivers on care within long-term care homes [46, 47, 67]. We were unable to conduct any meta-analyses due to heterogeneity across the included studies in outcomes, methodology, reporting, and data sources. Figure 2 presents a diagrammatic overview of included studies according to their study design and analytical approach. Figure 3 illustrates a synthesis of extracted data according to the three domains of Kilbourne *et al.’s* framework. There was evidence linking the contribution of domain factors to care-related outcomes at the resident level; most studies focussed on contributing factors found at the individual-level.

**Table 1:** Summary of all studies comparing care-related outcomes of long-term care home residents belonging to minoritized and context-specific dominant populations

	Contextual Characteristics						
Study	Data Timeframe	Country	Sample Size (n)	Age restriction of cohort	Outcome(s)	Study defined minoritised and dominant population	Main findings
Amir O. *et al.* [48]	2008–2009	United States	1,136,544	>65 years	Hip fracture	Minoritised: Native American, Black, Other Dominant: White	The crude incidence rate and the adjusted rate of hip fractures per 100 person-years remained higher among Native American [sic] residents compared to White [sic] residents.
Bates-Jensen *et al.* [49]	—	United States	142	>65 years	Pressure Injuries—developed during first 12 months of admission	Minoritised: Black, Asian, Hispanic Dominant: White	Mean number of pressure injuries were highest in Asian [sic] participants. Severity of pressure injuries were more severe in Black [sic] residents than other groups and they received prevention interventions at rates similar to other race/ethnicity groups.
Batista *et al*. [44]	2010–2016	Canada	47,727	≥50 years	Falls; Restraints; Pain management; Medication use/prescriptions.	Minoritised: Francophone Dominant: Anglophone	Descriptively, there were statistically significant differences across Francophones (Minority) [sic] and Anglophones (Majority), however, they did not remain significant after regression modelling
Baumgarten *et al.* [50]	1992–1995	United States	1,938	>65 years	Pressure Ulcers—the first occurrence of a Stage 2, 3, or 4	Minoritised: Black Dominant: White	Black [sic] residents had an unadjusted pressure ulcer rate that was 66% higher than that of white residents, hazard ratio was reduced from 1.66 to 1.35 for Black residents compared to White residents controlling for resident and home characteristics.
Bliss *et al.* [51]	2000–2002	United States	10,861	>65 years	Healing of Pressure Ulcers within 90 days of admission	Minoritised: American Indian, Alaskan Native, Asian/Pacific Islander, Black, Hispanic Dominant: White	The incidence of pressure ulcers for Black [sic] residents is greater and ulcers are less likely to heal by the 90-day MDS assessment for Black residents than would be expected had they been part of the White group.
Bliss *et al.* [52]	2000–2002	United States	90,575	>65 years	Pressure Ulcer Development and Treatment	Minoritised: American Indian/Alaskan Native, Asian/Pacific Islander, Non-Hispanic Black, Hispanic Dominant: White Non-Hispanic	The overall disparity in time to develop a pressure ulcer between Black [sic] and White Non-Hispanic [sic] admissions was 3% at 3 months and increased to 5.8% at 6 months and 7.7% at 18 months. There were no disparities in treatment of a pressure ulcer for any of the minority groups.
Bliss *et al.* [53]	2000–2002	United States	42,693	>65 years	Primary Prevention of Incontinence	Minoritised: American Indian/Alaskan Native, Asian/Pacific Islander, Black, Hispanic Dominant: White	The overall percent disparity in incontinence prevention was 2% and it was unexplained by predictors in the model for Black [sic] admissions.
Bliss *et al.* [54]	2000–2002	United States	42,693	>65 years	Incontinence	Minoritised: American Indian/Alaskan Native, Asian/Pacific Islander, Black, Hispanic Dominant: White	The proportion of Black [sic] residents expected to develop incontinence was like the proportion (34%) of White [sic] residents observed to do so, indicating no disparities exist across racial groups in the development of incontinence.
Bliss *et al.* [55]	2000–2002	United States	28,119	>65 years	Incontinence	Minoritised: American Indian/Alaskan Native, Asian/Pacific Islander, Black, Hispanic Dominant: White	Disparities in the time to cure of incontinence for Hispanic [sic] residents compared to Non-Hispanic White [sic] residents, however, there was no significant disparity in time to incontinence cure disadvantaging API [sic], AIAN, and Black [sic] admissions.
Bliss *et al.* [56]	2000–2002	United States	15,927	>65 years	Social Engagement	Minoritised: American Indian/Alaskan Native, Asian/Pacific Islander, Black, Hispanic Dominant: White	No racial or ethnic-based disparities in social engagement at 1 year were found. Overall risk factors for low social engagement were low social engagement at admission, deficits in activities in daily living and cognition, problems with vision and communication, and residing in a home in an urban community
Bliss *et al.* [57]	2000–2002	United States	10,713	>65 years	Incontinence associated skin damage (IASD) prevention	Minoritised: American Indian/Alaskan Native, Asian/Pacific Islander, Black, Hispanic Dominant: White	No disparity in IASD prevention was found across racial/ethnic groups. Predictors of prevention of IASD included a deficit in ADLs (OR: 1.02 [1.02–1.03]) and higher percents of residents on Medicaid (OR: 1.01 [1.00–1.02]), which could indicate both clinical indications and socioeconomic differences drive differences.
Bowblis *et al.* [58]	2015	United States	11,126	>65 years	Quality of Life	Minoritized: Non-Hispanic Black, Native American, Other Dominant: Non-Hispanic White	The results found QoL disparities between White [sic] and BIPOC [sic] residents. Across most QoL measures, resident characteristics explain less than 25.9% of the disparity. In contrast, differences in facility characteristics explain between 37.9% and 56.8% of the disparity for overall QoL and four domains.
13.Boyington et al. 2007 [59]	1999–2002	United States	95,911	>65 years	Urinary Incontinence	Minoritized: African Americans Dominant: Caucasian	The prevalence of urinary incontinence remained higher for African Americans [sic] at both time points than Caucasian [sic] residents. Even after controlling for different factors, there were higher odds for incontinence for African American [sic] men. This study highlights the need for facility-level analyses in the future.
Cai *et al.* [60]	2006–2007	United States	59,740	>65 years	Pressure Ulcers	Minoritised: Black Dominant: White	Black [sic] residents had higher odds of experiencing risk adjusted pressure ulcer outcomes than White [sic] residents in New York State nursing homes, which can be attributed to their disproportionate congregation in facilities with lower quality of care rather than within facility disparities.
Cai *et al.* [61]	2007–2010	United States	394,948	>65 years	Hospitalizations	Minoritised: Black Dominant: White	For dying residents without DNH orders and with no or mild cognitive impairment, Black [sic] residents had a slightly higher risk of EOL hospitalisation than White [sic] residents after accounting for individual characteristics and facility effects.
Christian *et al.* [62]	1992–1996	United States	19,051	>65 years	Medication use/prescriptions	Minoritized: American Indian, Asian/Pacific Islander, Non-Hispanic Black, Hispanic, Dominant: Non-Hispanic White	Non-Hispanic Black, Hispanic, and Asian/Pacific Islanders [sic] residents received less of secondary prevention agents than non-Hispanic whites. American-Indians [sic] received more of any preventive agent compared with non-Hispanic whites.
Gerardo *et al.* [63]	2000	United States	74,343	>65 years	Pressure Ulcers	Minoritized: Black, Hispanic. Dominant: non-Hispanic White	Hispanics and non-Hispanic Blacks [sic] had a higher prevalence of pressure ulcers than non-Hispanic Whites [sic]. Disparities in NH care clearly exist as all residents of NHs in which Hispanics reside are more likely to have a pressure ulcer. Further, Hispanics are more likely to have a pressure ulcer in NHs that house a greater share of Hispanics.
Grabowski *et al.* [64]	1998–2002	United States	—	Not reported	Restraints; Medication use/prescriptions; Catheters; Feeding tubes.	Minoritised: Black Dominant: White	There were meaningful disparities in terms of feeding tube use for Black [sic] residents compared to White [sic] residents, a rough doubling of the risk of feeding tube use after adjustment. No disparities in the other three observed process measures of quality after adjusting for controls.
Hanlon *et al.* [65]	2004–2005	United States	3,480	>65 years	Medication use/prescriptions	Minoritized: Black Dominant: White	Fewer Black [sic] residents received a selective serotonin reuptake inhibitor (SSRI) antidepressant or a second-generation antihistamine but more received opioids to White [sic] residents. No modelling was conducted.
Howard *et al.* [66]	1999–2002	United States	113,869	>65 years	Pressure ulcers	Minoritised: African American Dominant: White	Overall risk for pressure ulcers was higher for African American [sic] residents compared to White [sic] residents and varied by gender. For example, risk of pressure ulcers for female African American residents compared to female White residents was 28% higher among those who were bedfast. Estimated risk ratios were slightly higher in small nursing homes.
Kataoka-Yahiro *et al.* [67]	Not provided	United States	22	Not reported –Family members recruited	Family caregiver satisfaction with palliative care services	Minoritised: Japanese, Chinese, Filipino, Native Hawaiian/part Hawaiian Dominant: N/A	This study provided a description of Asians and Native Hawaiians [sic], family caregiver satisfaction with palliative care services provided by nursing home health professional staff. Findings suggest there is a need to address and maintain family caregiver and interdisciplinary staff communication based on culturally appropriate approaches and needs.
Kwak *et al.* [68]	2000–2002	United States	30,765	>65 years	Hospice use and in-hospital death	Minoritized: Non-Hispanic Black Dominant: White	Hospice use was overall low for all nursing home residents (28%). Black [sic] residents were significantly less likely to use hospice than Whites [sic] even after we controlled for other predisposing, enabling, and need factors and more likely to die in a hospital despite a powerful effect of hospice in reducing in-hospital death.
23.Li et al. 2011 [69]	2003–2008	United States	2446808	Not reported	Pressure Ulcers	Minoritized: Black Majority: White	Overall pressure ulcer rates decreased over years but Black [sic] residents showed persistently higher rates than white residents, even when adjusted patient demographic and clinical characteristics, home characteristics, by year, and by the concentration of Black residents.
Li *et al.* [70]	2012	United States	1,302,511	>65 years	Hospitalisations	Minoritised: Black Dominant: Non-Hispanic White	Black [sic] residents had higher risk for 30-day all-cause and potentially avoidable rehospitalizations than White [sic] residents. Such disparities persisted after adjustment for resident factors. All post-acute residents had higher risk for 30-day rehospitalization when admitted to skilled nursing facilities with higher proportions of Black resident admissions, but residents had similar rates of potentially avoidable re-hospitalisation within each group of skilled nursing facilities of similar Black resident concentrations.
Mack *et al.,* [71]	2011–2012	United States	342,920	≥50 years	Pain management	Minoritised: Non-Hispanic Black Dominant: Non-Hispanic White	Pain was common among residents; however non-Hispanic Black [sic] residents had less pain documented both self- and staff- reported than non-Hispanic White [sic] residents. Furthermore, irrespective of pain documentation, non-Hispanic Black residents were less likely to receive pain management than non-Hispanic White resident in nursing homes.
Mor *et al.* [72]	1995–1996	United States	15,640	Not reported	Hospitalisations	Minoritised: Black Majority: White	Both African American [sic] and White [sic] residents had a greater likelihood of being hospitalised in the last months of life when they resided in nursing homes with higher proportions of African Americans; however, a greater overall percentage of African Americans resided in facilities with a higher proportion of African Americans.
Morisson *et al.* [73]	2010–2016	United States	994,510	≥50 years	Pain management	Minoritised: Non-Hispanic Black, Hispanic Dominant: Non-Hispanic White	Non-Hispanic Black [sic] and Hispanic [sic] residents were less likely to receive any type of pharmacologic pain intervention compared to non-Hispanic White [sic] residents, even after controlling for demographic and clinical characteristics.
Rivera-Hernandez *et al.* [74]	2015	United States	1813963	>65 years	Hospitalizations	Minoritized: African American, Hispanic Dominant: White	There were statistically significant racial and ethnic differences of about 2 percentage points higher rates for African Americans [sic], and less than 1% for Hispanics [sic]. Although Medicare Advantage residents had lower readmission rates overall from fee-for-service residents, the racial/ethnic disparities were unchanged across groups.
Rosendahl *et al.* [46]	—	Western Europe/Sweden	—	Not reported –Family members/providers recruited	Adjustment to new living arrangements	Minoritised: Finnish, family [1], nurse [1], Estonian, nurse [1], Hungarian, family [1], nurse [1] Ingrian, family [1] Sweden, family [2], nurses [6]	The results of this study have reaffirmed that moving to a group home was a big change for people with dementia (PWD). However, adjusting to their new living arrangements varied among the immigrants with dementia.
Shippee *et al.* [75]	2010	United States	13,433	Not reported	Quality of Life	—	After accounting for resident status and health characteristics, racial differences in QoL became nonsignificant except for food enjoyment, with White [sic] residents reporting higher food enjoyment scores than their non-White [sic] counterparts.
Shippee *et al.* [76]	2011–2015	United States	60,093	Not reported	Quality of Life	—	QoL summary scores for White [sic] respondents were stable at about 80 points but declined for minority respondents from 75.9 points in 2011 to 73.7 points in 2015. Adjusting for individual covariates did not appreciably reduce the disparity, but including facility characteristics resulted in narrowing, but not eliminating the disparity.
Strandroos *et al.* [47]		Sweden	19	Not reported –Family members/providers recruited	Advantages of shared spoken language	Minoritised: Linguistic minorities: Finnish [5], Hungarian [1], Polish [1] Kurdish [1] Arabic [1]. Dominant: Swedish [9]	Although this study displays that shared language can be advantageous for residents in nursing homes, there is not sufficient evidence to explain this finding further.
Temkin-Greener *et al.* [77]	2014–2017	United States	665,033	Not reported	End-of-life hospitalizations	Minoritised: Black Dominant: White	In the last 30 days of life, Black [sic] residents with Alzheimer’s disease and related dementias (ADRD) who live in nursing homes experience significantly higher risk of hospitalizations compared to White [sic] residents. EOL hospitalisation risk remains significantly elevated for Black residents with ADRD and severe cognitive impairment. Disparities between black and white nursing home residents with ADRD, in EOL hospitalisations, persist both within and across facilities.
Yap *et al.* [45]	—	United States; Canada	505	>65 years	Pressure Ulcers	Minoritised: Asian Dominant: Non-Asian	This study points to the importance of protein dietary intake in relation to Pressure Injuries prevention. However, no modelling was conducted, and further study is indicated.

### Risk of bias assessment

Nine studies had a low risk of bias [51, 52, 54, 56, 57, 63, 69, 75, 77]. More than half (52.9%) of the studies had a moderate risk of bias due to a lack of information on missing data [48, 64, 68, 70, 72–74], small sample size [49], potential confounding [50, 58, 60, 70, 71, 73, 74, 76], a lack of multi-level modelling to account for clustering effects [59, 61, 62], outcomes not explored across all subgroups [55, 62, 64, 71] and potential misclassification [70, 76]. Four studies were at a severe risk of bias for potential confounding [45, 53, 65, 66], due to a lack of information on outcome assessment [53], a lack of information on missing data [65] and the outcome measurement not explored across all subgroups [65]. The risk of bias assessments is also summarised according to whether they included individual, provider and/or healthcare system factors in Table 2.

**Table 2:** Risk of bias assessment results for included quantitative studies

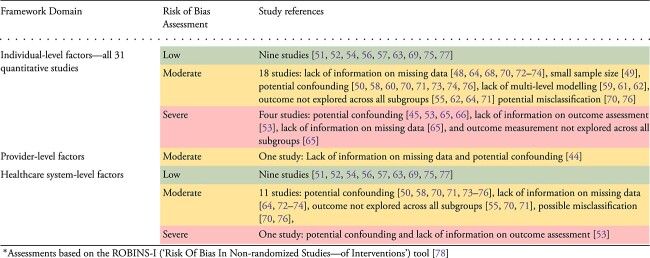		

### Care-related outcomes

Care-related outcomes examined in the quantitative studies (Appendix III) included pressure ulcers (*n* = 9) [45, 49–52, 60, 63, 66, 69], hospitalisation (n = 6) [61, 68, 70, 72, 74, 77], incontinence (*n* = 5) [53–55, 57, 59], quality of life (*n* = 4) [58, 67, 75, 76], pain (*n* = 2) [71, 73], restraints (*n* = 2) [44, 64], psychiatric diagnosis and antipsychotic prescriptions (*n* = 2) [44, 64], pharmaceutical interventions (*n* = 1) [65], secondary stroke prevention (*n* = 1) [62], hospice use (*n* = 1) [68], hip fractures (*n* = 1) [48] and social engagement (*n* = 1) [56]. Some studies (*n* = 4) reported more than one care-related outcome [44, 52, 64, 70].

The qualitative studies focused on the provider and resident experience [56]. Specifically, the experiences of immigrants to Finland living with dementia from the perspective of family members and nurses [46], Asian and native Hawaiian family caregiver satisfaction with care [67], and the perspectives of healthcare workers and persons living with dementia on challenges with communication across linguistic and cultural diversity [47].

### Individual-level resident factors

Individual factors that may contribute to health disparities in care-related outcomes include demographics, social accessibility, familial and cultural context and genetics [7]. We described characteristics of individuals within each cohort to provide context in understanding underlying determinants of health that contribute to care-related health disparities.

All quantitative studies found individual-level baseline differences between context-specific dominant and minoritised populations in the unadjusted descriptive results. Consistent differences in demographic and health characteristics (e.g. age, sex, education level, Medicaid funding and chronic conditions) and care-related characteristics (e.g. tube-feeding, restraint use and number of medications) associated with observed outcome disparities, and the direction of their associations are summarised in Appendix IV.

Seven studies (23%) modelled the relationship between care-related outcomes and the minoritised identity of interest, controlling for confounding covariates using single-level regression models (without accounting for clustering effects) [44, 48, 59, 61, 62, 66, 68]. Three of these studies included facility-level characteristics in the same model (i.e. at the same level) as individual resident characteristics [44, 48, 66]. Six found significant differences in care-related outcomes between minoritised populations and the dominant populations, after controlling for confounding factors [48, 59, 61, 62, 66, 68]. Two studies only provided descriptive statistics and did not control for confounding covariates in a model [45, 65].

### Provider factors

Provider factors that may contribute to health disparities include stereotyping, communication problems and a lack of cultural competence [7]. Providers impact a group of individuals (i.e. their patients and colleagues) and the identification of provider characteristics associated with health disparities could help focus interventions and policies at the organisational level.

A Canadian quantitative study on the association between linguistic discordance in long-term care homes and residents’ outcomes suggested patient-provider language may influence reporting of pain and restraint use. However, the results from this study did not remain significant after covariate adjustment. Additionally, language data used in this study was not validated, and the study is at a moderate risk of bias [44].

The three qualitative studies also primarily focused on residents who were identified as linguistic minorities. They highlighted provider-level issues that impacted the clinical encounter and may subsequently affect residents’ outcomes [46, 47, 67]. Two of these studies interviewed caregivers and family [46, 67], one of which included interviews with staff caring for persons living with dementia [46]. The third used ethnographic methodology, namely participant observation, and also included interviews with staff caring for those living with dementia [47]. The qualitative findings suggested language concordance between providers and family caregivers was helpful in supporting nuanced communication and preventing misunderstandings [46]. However, other aspects, such as continuity of interpersonal relations, the use of objects and the physical environment, were equally important [47]. The third study was on Asian and native Hawaiian [sic] family caregiver satisfaction with palliative care services [67]. This study identified the following themes: patient physical symptoms and comfort, information, family support and patient psychological care. They reported more culturally appropriate approaches to long-term care home staff communication are needed to address the needs of Asian and native Hawaiian residents.

### Healthcare system-level factors

Healthcare system level factors that may contribute to health disparities include types of remuneration model for services providers, care coordination and distribution of health human resources across the care continuum [7]. We described characteristics and methodological approaches at the level of healthcare organization to highlight areas contributing to inequity or advancing equity.

A total of 29 studies (94%) used interfacility data [44, 48, 50–66, 68–77, 79]; of these, 7 did not adjust for facility-level characteristics or included them within the same single-level model as resident-level characteristics. The majority (75.9%, *n* = 22) of these studies controlled for facility-level characteristics using clustered analysis with hierarchical fixed- or random-effects models to account for confounding attributable to unobserved home-level characteristics [51–58, 60, 61, 63, 64, 66, 69–77]. Fifteen studies found higher rates and proportions of disparities among minoritised populations, compared to the context-specific majority population, after controlling for both individual demographics and facility characteristics [50–53, 55, 58, 60, 63, 64, 69, 71, 73, 74, 76, 77]. Two studies analysed individuals as well as homes by the percentage of residents belonging to a minority population, both found homes with higher proportions of minoritised residents were more likely to report poorer outcomes at the aggregate level, even after controlling for several confounding characteristics [60, 72]. Four studies found observed differences in descriptive statistics were not statistically significant after modelling with confounding covariates [44, 56, 57, 75].

## Discussion

Evidence summarised in this review suggests that disparities in care-related outcomes exist across populations residing in long-term care homes. Specifically, minoritised populations are entering long-term care homes with baseline differences in individual-level demographic and health characteristics from majority populations. Baseline differences may contribute to disparities in care-related outcomes experienced at the time of admission into the facility—with higher reported rates of poor outcomes among minority populations—and throughout care within the facilities, with evidence that disparities persist even after adjusting for confounding variables. One key observation made in several studies is the variation in health care and outcomes across facilities. This suggests quality of care is often not uniform across homes within regions. In particular, findings from ecological studies suggest that greater proportions of those belonging to minoritised populations reside in homes with lower standards of care. Still, caution should be taken when drawing conclusions as there is heterogeneity across the studies, a moderate risk of bias in a large proportion of both individual- and system-level evidence, and few studies accounted for underlying determinants of health and structural system-level factors.

### Interpretation

Findings from this review demonstrate individual- as well as organisational- and healthcare system-level factors interact and intersect to influence the outcomes of clinical encounters among minoritised individuals. Untangling the role of the various determinants of health disparities may be achieved in research by applying relevant theoretical or conceptual frameworks to guide the design of research studies—from variable selection (i.e. ensuring pertinent patient and provider characteristics, as well as system factors are captured) to modelling strategies that account for clustering effects.

An example that illustrates the importance of accounting for dynamic, multilayered individual, group and system-level factors is the experience of long-term care recipients supported by the U.S. Medicaid program, a federal program that offers partial coverage of medical fees to individuals with low income. Minoritised older adults in the United States, where most of the review evidence originates, are more likely to experience systemic barriers to healthcare and have characteristics associated with poorer care-related outcomes (e.g. lower income, enrolled in the Medicaid program and receiving care where a high proportion of other residents are also Medicaid recipients) [80, 81]. Previous evidence suggests quality of care and resident characteristics are associated [82–85]. However, this relationship may be mediated by structural variations experienced by groups of individuals with shared characteristics; specifically, the higher prevalence of adverse outcomes in long-term care homes with larger proportions of residents belonging to racial and ethnic minority populations [86–89]. This suggests heterogeneity in facility-level quality may be responsible for disparities and rather than differential treatment of those belonging to a minority population within these homes. In other words, a poorly specified statistical model not fully accounting for clustering effects at organisational levels may modify or hide the true burden of health disparities, and misattribute the effects to individual variations.

All the U.S. based studies categorised minority populations according to residents’ race and ethnicity which has several historical and political indications. Although identifying and addressing disparities is essential to improving health equity, research on minoritised communities has been cautioned to consider how the use of racial categories is misinformed [90, 91] and could reinforce socially constructed ‘othering’ of groups marginalized by society. Instead, researchers are encouraged to seek equitable solutions that address unique factors contributing to inequities [92, 93]. We urge those engaging in this research to understand factors influencing differences in care and, where possible, collect relevant data that will not perpetuate racism [91, 94]. For example, collecting and using data on characteristics of minoritised populations (e.g. race) without exploring the intersection of these characteristics with other aspects of minoritised individuals’ experiences—including economic, social and environmental differences—is likely insufficient. The intersection of identities [95, 96] is important to address as these factors and their relationships that may explain, mediate or confound the relationship between minority status and care-related outcomes.

Finally, there is a significant paucity of international evidence on care-related outcomes for minoritised populations living in long-term care, with variation in study design and outcome measurement. Existing evidence on provider-level factors relating to care-related outcomes is largely qualitative, highlighting a need for ongoing provider-level data collection including in health administrative data sets. For example, a 2017 U.S. study found a higher ratio of Licensed Practical Nurses to Registered Nurses resulted in higher odds of readmission to hospital and mortality for skilled nursing facility residents [97]. Several populations and outcomes had minimal or no evidence, notably several ethnic groups, Indigenous peoples, religious and spiritual groups, those who identify as part of the 2SLGBTQIA+ community (abbreviation for: Two-Spirit, Lesbian, Gay, Bi-sexual, Trans, Queer and/or questioning, Intersex, and Asexual) [98], or those living with a disability (including invisible and episodic disabilities). Future research could explore care-related outcomes among more minoritised populations using conceptual designs and modelling techniques that account for the intersection of contributing factors.

### Strengths and limitations

The strength of this review is that it is the first to summarise evidence on care-related outcomes of minoritised populations living within long-term care and highlight key findings and knowledge gaps. It is also the first to contextualise evidence on minoritised populations within an existing framework that supports advancing health equity without reinforcing marginalization. However, there are limitations to consider. We only searched for indexed articles published in the English or French language and therefore may have missed international work. There was significant heterogeneity across the included studies in methodology, reporting and data sources which resulted in a higher-level data synthesis and inconclusive findings on the specific reported associations. Finally, the evidence summary was mostly inconclusive due to the inability to conduct a meta-analysis or pooled estimate and little to no evidence on some known ageing minoritised groups that require this care (i.e. Indigenous peoples and 2SLGBTQIA+ communities) and the intersection of minoritised identities and characteristics.

## Conclusion

Our results found distinct differences across several minoritised groups entering and living within long-term care homes. By highlighting the origins of disparities, this review aims to bring a renewed focus on providing equal support of those entering and living within long-term care based on their level of need, while acknowledging the existence of structural vulnerabilities that impact the care and outcomes of many minoritised groups. This work aims to advance health equity by directing future research and policy to integrate the multiple dynamic layers into methods and decisions on best practices, rather than continuing to describe persistent inequality through the lens of those with a disadvantaged experience.

## Supplementary Material

aa-23-0681-File002_afae059

## Data Availability

The data that support the findings of this study are available from the corresponding author, upon reasonable request.
