# Supplementary material for: Building evidence to advance health equity: a systematic review on care-related outcomes for older, minoritised populations in long-term care homes
Source: Age Ageing. 2024 Apr 1;53(4):afae059. doi: 10.1093/ageing/afae059 (PMC10982852; doi:10.1093/ageing/afae059)
Supplement: aa-23-0681-File002_afae059 [file aa-23-0681-file002_afae059.docx]

**Building evidence to advance health equity: a systematic review on care-related outcomes for older, minoritized populations in long-term care homes.**

**Supplementary Materials Contents List**

Appendix I: Search strategy

Appendix II: Detailed description of methodologies applied in data screening and extraction, risk of bias assessment, and data synthesis

Appendix III: Results summarized according to the care-related outcome of the included quantitative studies

Appendix IV: Selected baseline differences in minority populations compared to majority populations entering or residing within long-term care

**Appendix I: Search strategy**

Database: Ovid MEDLINE(R) ALL <1946 to September 03, 2021>

Search Strategy:

--------------------------------------------------------------------------------

1 exp ethnic groups/ (163729)

2 (ancestry or continental).hw. (180074)

3 population groups/eh (623)

4 "emigrants and immigrants"/ (13502)

5 (emigrant* or immigra* or migrant* or newcomer* or refugee*).tw,kf. (65731)

6 minority groups/ (15277)

7 minority health/ (847)

8 (minorities or minority group* or minority health*).tw,kf. (20721)

9 alaska natives/ or american native continental ancestry group/ or indians, north american/ or inuits/ (18107)

10 (aborigin* or eskimo* or first nation* or indian* or indigenous or innu or inuit* or inuk* or maori* or metis or native* or tribal or tribe*).tw,kf. (373764)

11 (native* adj (alask* or american* or canadian* or Hawaii*)).tw,kf. (7063)

12 oceanic ancestry group/ (11084)

13 ((pacific or torres strait) adj islander*).tw,kf. (6015)

14 (american adj (indian* or samoan*)).tw,kf. (7399)

15 ((african or asian or black or blacks or ethnic* or hispanic* or latina* or latino* or latinx or middle eastern or "of color" or "of colour" or race or racial*) adj25 (adult* or elder* or men or older or patient* or people* or population* or retire* or resident* or senior citizen* or wom*n)).tw,kf. (277493)

16 (acadian* or acadien* or allophone* or bilingual or ESL or francophone* or francoontar* or franco-ontar* or french canadian* or french-speak* or language* or linguistic* or quebecois* or quebecker*).tw,kf. (198355)

17 (crosscultural or cross-cultural or intercultural or inter-cultural or multicultural or multi-cultural).tw,kf. (18888)

18 exp "sexual and gender minorities"/ (9736)

19 (bipoc or bisexual* or gay? or homosexual* or lesbian* or lgbt* or "men who have sex with men" or non-binar* or queer* or same-sex* or transm*n or transwom*n or transgender* or two-spirit* or "wom*n who have sex with wom*n").tw,kf. (457285)

20 or/1-19 (1498912)

21 residential facilities/ (5639)

22 assisted living facilities/ (1499)

23 long-term care/ (26940)

24 exp nursing homes/ (41574)

25 homes for the aged/ (14462)

26 ((assisted living or care or nursing or residential or retirement or skilled nursing) adj2 facilit*).tw,kf. (36598)

27 ((care or nursing or retirement) adj home*).tw,kf. (37377)

28 ((elder* or extended or intermediate or longterm or long term) adj2 (care* or healthcare)).tw,kf. (37317)

29 (ltc or ltcf).tw,kf. (4612)

30 (end-of-life adj2 (care* or treat*)).tw,kf. (13478)

31 or/21-30 (143427)

32 healthcare disparities/ (19615)

33 health status disparities/ (17867)

34 ((ethnic or health*) adj2 disparit*).tw,kf. (25196)

35 race factors/ (614)

36 ((race or racial) adj8 (aspect* or factor or factors)).tw,kf. (10886)

37 ((cultural or ethnic* or religious or socio-economic) adj2 (aspect* or factor or factors)).tw,kf. (14426)

38 health equity/ (2069)

39 culturally competent care/ (1860)

40 ((crosscultural or cross-cultural or intercultural or inter-cultural or transcultural or trans-cultural) adj2 (care or health*)).tw,kf. (738)

41 cultural competency/ (6022)

42 ((cultur* or language* or linguistic*) adj2 (appropriate* or competenc* or congru* or discord* or incongru* or sensitiv*)).tw,kf. (22157)

43 ("at risk" adj2 (group* or populat* or resident*)).tw,kf. (18610)

44 ((barrier* or bias or burden* or constraint* or equit* or factor* or fairness or inclusion or inclusiv* or inequal* or inequit*) adj3 (care or health or healthcare or ltc or ltcf or quality or referral* or treating or treatment*)).ti,kf. (31966)

45 ((barrier* or bias or burden* or constraint* or equit* or factor* or fairness or inclusion or inclusiv* or inequal* or inequit*) adj15 (care or health or healthcare or ltc or ltcf or quality or referral* or treating or treatment*)).ab. /freq=2 (167693)

46 ((challeng* or differ* or disadvant* or discriminat* or disparities or disparity or influenc* or obstacle* or persecut* or prejudic* or segregat*) adj3 (cultural* or diverse or diversity or ethnic* or gender* or language* or linguistic* or nationalit* or racial* or religion* or religious* or sex*)).ti,kf. (68638)

47 ((challeng* or differ* or disadvant* or discriminat* or disparities or disparity or influenc* or obstacle* or persecut* or prejudic* or segregat*) adj10 (cultural* or diverse or diversity or ethnic* or gender* or language* or linguistic* or nationalit* or racial* or religion* or religious* or sex*)).ab. /freq=2 (104609)

48 ((asian or asians or black or blacks or latina* or latino* or latinx or indigenous or hispanic* or "of color" or "of colour") and (white or whites or caucasian*)).tw. (90578)

49 or/32-48 (484082)

50 20 and 31 and 49 (2539)

51 limit 50 to yr="2000 -Current" (2326)

**Appendix II: Detailed description of methodologies applied in data screening and extraction, risk of bias assessment, and data synthesis**

Screening and extraction

Title and abstracts and full text articles were screened by two independent reviewers using Covidence software.^41^ Extraction was conducted by one author, independently using a pre‐tested form, with a second author verifying the extracted information. Conflicts and discrepancies were discussed with a third author, if required to reach consensus.

Risk of Bias Assessment

The ROBINS-I (Risk Of Bias In Non-randomised Studies of Interventions) tool was used to assess the risk of bias in the quantitative studies.^42^ Each risk of bias decision was extracted and reviewed by a second author. Conflicts were reviewed and discussed until a consensus was reached.

*Synthesis*

The studies were first organized and summarized according to each care-related outcome (e.g., incontinence, pain, pressure ulcers) and subsequently according to the conceptual framework proposed by Kilbourne et al.^7^ The authors assessed the plausibility of a meta-analysis, a method of summarizing homogeneous evidence to determine an absolute effect across studies.^43^

|  | | |
| --- | --- | --- |
| **Appendix III: Results summarized according to the care-related outcome of the included quantitative studies** | | |
| **Outcome** | **Risk of Bias Assessment*** | **Reasons for bias and study references** |
|  |  |  |
| Pressure Ulcers | Low | Seven studies: ^48,53,55,57,58,69,71,80,101,102^ |
|  | Moderate | Three studies: potential confounding^69,71^ and small sample size^68^ |
| Hospitalizations | Low | One study: ^60^ |
|  | Moderate | Five studies: lack of information on missing data,^63-65,67,75^ potential confounding,^39,64,67^ and possible misclassification^64,75^ |
| Incontinence | Low | Three studies: ^54,56,74^ |
|  | Moderate | One study: outcome not explored across all subgroups^77^ |
|  | Severe | One study: potential confounding and lack of information on outcome assessment^78^ |
| Quality of life | Low | One study:^59^ |
|  | Moderate | Two studies: Potential confounding,^70,73^ potential misclassification.^73^ |
| Pain | Moderate | Two studies: Potential confounding,^66,72^ lack of information on missing data,^66^ outcome not measured across all subgroups.^72^ |
| Restraints | Moderate | Two studies: Potential confounding,^47,62^ lack of information on missing data,^47^ and outcome measurement not explored across all subgroups.^62^ |
| Psychiatric diagnosis and antipsychotic prescriptions | Moderate | Two studies: Potential confounding,^47,62^ lack of information on missing data,^47^ and outcome measurement not explored across all subgroups.^62^ |
| Pharmaceutical interventions | Severe | One study: potential confounding, lack of information on missing data, and outcome measurement not explored across all subgroups^79^ |
| Secondary stroke prevention | Moderate | One study: Outcome measurement not explored across all subgroups ^76^ |
| Hospice use | Moderate | One study: lack of information on missing data^63^ |
| Hip fractures | Moderate | One study: lack of information on missing data.^61^ |
| Social Engagement | Low | One study:^52^ |

*Assessments based on the ROBINS-I (“Risk Of Bias In Non-randomised Studies - of Interventions”) tool^42^

**Appendix III Cont. - Narrative summary of results by care-related outcomes**

**Pressure Ulcers**

Pressure ulcers are tissue damage created by prolonged pressure, often occurring in residents who are immobile that are not being routinely moved. Pressure ulcers are assessed according to four stages, beginning with stage I, in which skin redness does not disappear after pressure is removed, to stage IV with tissue loss exposing muscle and bone.

Three studies reported incidence rates of pressure ulcers,^48,69,101^ four reported the prevalence of pressure ulcers,^53,57,58,68^ two reported on risk of developing pressure ulcers,^71,80^ and one measured the healing of pressure ulcers within 90 days of admission.^55^ All studies reported higher rates, higher risk, and higher prevalence of pressure ulcers in at least one of the racial minorities compared to the White [sic] majority. Furthermore, Black [sic] populations were more likely to have advanced stages of pressure ulcers^80,101^ and were less likely to heal within 90 days of admission compared to white majority populations.^55^ One study found that despite having higher prevalence, the rate that Black residents received prevention interventions were similar to other racial and ethnic groups.^68^

Rosen et al. was the only study to introduce an educational intervention designed to increase staff awareness of preventing pressure ulcers.^101^ The incidence of pressure ulcers measured at baseline found Black residents were more likely to have multiple stage II pressure ulcers and less likely to have a stage I ulcer identified compared to White residents. After the intervention was introduced, rates of pressure ulcers were reduced and racial disparity was eliminated.

**Hospitalizations**

Hospitalization is commonly used as an indicator of care quality and patient-centered outcomes.^103^ Hospital admissions for those at the end of life can be burdensome and may contribute to patients’ reduced quality of life, particularly readmissions within 30 days of a hospital discharge.^104^ Authors of this review note there are challenges with interpreting this outcome as critically ill patients may benefit from a hospitalization to stabilize or treat their condition, especially when resources are not available in their home environment.

Six studies reported hospitalizations^60,63-65,67,75^ all of which were US based.^60,63-65,67,75^ Two reported all cause 30 day readmission,^64,67^ and three were on end-of-life hospitalizations.^60,65,75^ One study measured in-hospital deaths for nursing home residents.^63^ Rehospitalization rate within 30 days of discharge was higher for Black [sic]^64,67^ and Hispanic [sic]^67^ residents compared to White [sic] residents. The rate of end-of-life hospitalizations was higher for Black residents,^60,75^ with significantly higher risk among those living in facilities with higher proportions of minority population^65^ after controlling for resident and facility characteristics.^60^ Kwak et al. found Black residents were 80% more likely to die in a hospital than White residents, even after adjusting for covariates, including hospice use.^63^

**Incontinence related outcomes**

Incontinence is loss of toileting control and can be a result of not receiving timely help to relieve oneself.

Five studies reported incontinence. All five studies were based in the US, included participants >65 years old, and defined minorities based on race or ethnicity.^54,56,74,77,78^ Each study measured a different outcome related to incontinence including prevalence,^74^ primary prevention treatment,^78^ risk of developing incontinence,^54^ risk of developing incontinence associated skin damage,^56^ and measuring time to cure incontinence.^77^ Overall prevalence of incontinence increased overtime, however was higher in African Americans [sic] compared to Caucasians [sic], with risk remaining significant after model adjustment. A 2% disparity in primary prevention treatment of incontinence was observed in Black [sic] compared to White [sic] residents, with no disparities across other racial minorities.^78^ The risk of developing incontinence-related skin damage and the risk of developing incontinence was not significantly different between minority and majority populations after adjusting for individual and facility level characteristics.^54,56^ Time to cure for Hispanic [sic] residents was longer and a smaller proportion of Hispanic residents were cured compared to white residents, however significant differences were not found for other racial minorities.^78^

**Quality of life**

Quality of life is an outcome that is related to care, along with other patient-centred factors including their care needs and level of independence, the amount of unpaid support, and their social engagement.

Quality of life was reported in four studies which were all US-based,^59,70,73^ one study restricted age to those >65 years,^70^ one was qualitative,^51^ and all defined minorities based on race or ethnicity, however two combined racial minorities together and compared them to white majority.^59,73^

Two studies measured quality of life across six domains: environment, personal attention, food, engagement, negative mood, and positive mood.^59,70^ One study found that facility characteristics explained 92.7% of the disparity in environment and negative mood, while only 37.9% of the overall quality of life score.^70^ All three quantitative studies reported a summary quality of life score and measured differences according to both individual- and facility-level factors.^59,70,73^ All studies found that facility-level characteristics explained variations in quality of life better than individual-level characteristics, with one reporting only 5% of the variation was explained by individual-level characteristics.^70^ Two facility-level analyses were ecology-based, with categorization of homes according to the composition of residents belonging to a racial minority. Both studies found a higher score of quality of life was associated with homes that housed mostly White residents [sic], the racial majority in the U.S.^59,73^

**Pain**

Pain is a difficult measure to assess given its subjective nature making establishing a baseline benchmark challenging. However, pain is often able to be managed through care interventions.

Three studies on pain in long-term care residents were included and each reported on the prevalence of pain.^47,66,72^ Two U.S. based studied found Black residents were less likely to be treated for pain.^66,72^ One found Hispanic residents were more likely than Black residents to have pain treated but were still more likely to have undertreated pain compared to White residents. ^66^ The one Canadian study determined linguistic concordance according to the proportion of residents with a common language and found Francophone residents were more likely to report pain than Anglophones, particularly in linguistically concordant homes, however the discrepancy did not persist after adjusting for individual-level and facility-level characteristics.^47^

**Restraints**

Restraints are physical interventions defined as “any device (for example, a physical or mechanical device, material or equipment attached or adjacent to the resident’s body) that the resident cannot easily remove and that restricts freedom of movement or normal access to their body.” Many homes minimize the use of restraints as much as possible, with the aim of supporting residents to have more independence. They are also understood to be associated with lower quality of life and could be indicative of low care quality due to inadequate staffing or neglect.

Two studies reported restraint use. One study was US-based and defined minorities according to race and ethnicity.^62^ The other study was based in Canada and explored outcomes of Francophone linguistic minorities in language discordant environments.^47^ Higher proportions of residents belonging to a minority population were restrained and restraints were reported as a secondary outcome.^47,62^ They both found no statistically significant difference in restraint use between minority and majority populations after adjusting for covariates,^47,62^ however higher rates of physical restraint use was observed in Canadian French-designated facilities.^47^

**Psychiatric diagnosis and antipsychotic prescriptions**

Diagnosis of mood-related conditions and subsequent treatment of these conditions are included as a care-related outcome as the prevalence of such conditions are known to be high. Diagnosis and treatment may be indicative of attentive, high-quality care. Appropriate prescribing in long-term care is an important aspect of care that impacts residents’ symptom management and well-being.

Two studies reported on psychoactive treatments.^47^ One US-based study measured the provision of anti-psychotic medication and the other was a Canadian study on care quality for Francophone and Anglophone residents in linguistically discordant and concordant environments.^47,62^ Grabrowski et al. found that although anti-psychotic medication was lower in Black residents compared to White residents [sic], association between race and care quality did not remain after controlling for individual-level and facility-level characteristics and including a mediation analysis.^50^ Batista et al. found a smaller proportion of Francophones in French-designated facilities were prescribed antipsychotics despite not having a diagnosis of psychosis, experienced worsening depressive symptoms, and experienced fewer falls compared to Francophones in non-designated facilities. After adjusting for confounding, these trends disappeared and no statistically significant difference was observed.^47^

**Pharmaceutical interventions**

Medication use was measured in one US-based study across racial categories: Black [sic] residents compared to White residents [sic].^79^ The study found Black residents were less likely to receive central nervous system (CNS) medications and antihistamines, however were more likely to receive opioids than White residents. This analysis was purely descriptive based on the significant difference in proportions of medication use across Black and White residents and did not model the relationship through further statistical analyses.

**Preventative stroke care**

Strokes are a common acute ischaemic or haemorrhagic vascular event resulting in neurological deficit. They are more common in older adults and can be debilitating with a high risk of mortality, with even higher risks found among certain minority populations.^105^ There are preventative measures to help treat individuals at risk. The incidence of initiating and discontinuing preventative stroke care could indicate care quality.

One study assessed secondary stroke prevention across racial groups in U.S.-based nursing homes.^76^ The absolute difference in prevalence of receiving warfarin (a medication commonly used to prevent stroke) amongst those eligible, was found to be lower in Asian/Pacific Islanders, non-Hispanic Black residents, and Hispanics compared to non-Hispanic White residents [sic]. After control of confounding, all minority groups eligible for anticoagulant therapy received warfarin less often than non-Hispanic White residents [sic].

**Hospice use**

Hospice - founded in England during the 1960’s - is care specifically designed to provide specialized end-of-life care for those facing life-limiting illness.^106^ There are a number of models that have been implemented internationally, with concerns that culturally relevant hospice care is not provided to minority populations.^107,108^

One study was on hospice use conducted in the U.S.^63^ Comparing the prevalence of hospice use and assessing the association with predisposing, enabling, and need factors across Black and White residents [sic], the overall prevalence of hospice use was low (28%). Black residents were significantly less likely to use hospice than white residents, even after controlling for a number of factors.^63^

**Hip fracture**

Hip fractures could be related to advanced frailty and often a fall-related injury. Many interventions are used in long-term care or nursing homes to prevent these injuries.

This U.S.-based study was on the incidence rates of hip fracture among Native Americans [sic] compared to all other racial groups.^61^ The unadjusted incidence of hospitalized hip fracture was higher amongst Native American residents compared to the overall proportion. After adjustment, using inverse probability weighting, the incidence rate of hospitalized hip fracture was highest for Native American residents followed by white, other [sic] racial groups, and Black [sic] residents.

**Social engagement**

Promoting social engagement is an important component of care for older adults living in long-term care homes. Low social engagement is associated with reduced quality of life, loneliness, and increased mortality rates.^109^

One study from the U.S. assessed whether there were racial and ethnic disparities in social engagement within long-term care residents at one year after admission.^52^ No disparities in social engagement between minority and majority racial and ethnic groups were found, however other factors, including living in an urban home and physical limitations were associated with a higher risk of low social engagement at one year after admission.

**Appendix IV: Selected baseline differences in minority populations compared to majority populations entering or residing within long-term care**

| **Baseline Characteristics** | **Direction of the difference for minority populations* (% or mean)** | **Number of studies** | **Study references** |
| --- | --- | --- | --- |
| Younger age | ↑ | 17 | ^57-64,66,67,69-73,79,80^ |
| Male sex | ↑ | 11 | ^57-60,62,64,66,71,72,75,80^ |
| Lower education level | ↑ | 10 | ^52-54,56,62,75,77-80^ |
| Medicaid insured | ↑ | 6 | ^59,62,64,67-69^ |
| Average number of medications | ↓ | 6 | ^52,54,55,75,77,78^ |
| Bedfast | ↑ | 6 | ^53,55,57,69,74,80^ |
| Using a feeding tube | ↑ | 7 | ^52,53,55,56,62,77,78^ |
| Diagnosed diabetes | ↑ | 10 | ^57,58,67-69,71,75,76,80^ |
| Diagnosed depression | ↓ | 11 | ^52-54,56,60,71,73,75,77-79,110^ |
| Diagnosed cancer | ↑ | 6 | ^60,63-65,75,79^ |
| Communication difficulty | ↑ | 5 | ^52,53,55,64,78^ |

*differences in p-values <0.5% or proportions ≥1%
